# Genotypic and phenotypic characteristics of *Acinetobacter baumannii* isolates from the people’s hospital of Qingyang City, Gansu province

**DOI:** 10.1186/s12864-024-10601-x

**Published:** 2024-07-26

**Authors:** Jiali Chen, Yang Wang, Na Zhang, Juan Li, Xiong Liu

**Affiliations:** 1 Chinese PLA Center for Disease Control and Prevention, Beijing, 100071 China; 2 School of Medicine, Nankai University, Tianjin, 300071 China; 3 State Key Laboratory of Mycology, Institute of Microbiology, Chinese Academy of Sciences, Beijing, 100101 China; 4 University of Chinese Academy of Sciences, Beijing, 100101 China; 5 Department of Clinical Laboratory Medicine, Qingyang People’s Hospital, Qingyang, Gansu, 745000 China

**Keywords:** *Acinetobacter baumannii*, Multi-drug resistance, Whole-genome sequencing

## Abstract

**Background:**

*Acinetobacter baumannii* (*A. baumannii*) is a common opportunistic pathogen in hospitals that causes nosocomial infection. In order to understand the phenotypic and genotypic characteristics of *A. baumannii* isolates, we sequenced and analyzed 62 *A. baumannii isolates* from a hospital in Gansu province.

**Results:**

Non-repeated 62 *A. baumannii* isolates were collected from August 2015 to November 2021. Most isolates (56/62) were resistant to multiple drugs. All the 62 *A. baumannii* isolates were resistant to aztreonam and contained *bla*_ADC-25_ gene which exists only on chromosome contigs. The 62 isolates in this study were not clustered in a single clade, but were dispersed among multiple clades in the common genome. Seven sequence types were identified by Multilocus sequence type (MLST) analysis and most isolates (52/62) belonged to ST2. The plasmids were grouped into 11 clusters by MOB-suite.

**Conclusions:**

This study furthers the understanding of *A. baumannii* antimicrobial-resistant genotypes, and may aid in prevention and control nosocomial infection caused by drug-resistant *A. baumannii*.

**Supplementary Information:**

The online version contains supplementary material available at 10.1186/s12864-024-10601-x.

## Background

*Acinetobacter baumannii* (*A. baumannii*) is a Gram-negative opportunistic pathogen, which can cause pneumonia, meningitis, wound infection, urinary tract infection, bacteremia and so on [1]. In all kinds of hospital-acquired infections caused by Gram-negative bacteria, *A. baumannii* accounted for 2%-10% [2, 3]. According to the declaration from the World Health Organization (WHO), *A. baumannii* has become one of the most threatening pathogens and an important focus of public health field and clinical research [4].

In recent years, the infection and drug resistance rates of *A. baumannii* have been increasing due to various factors, including the widespread use of antibiotics, the treatment of related immunosuppressants and invasive intervention. Some studies show that *A. baumannii* has the highest multidrug resistance (MDR) and extensive drug resistance (XDR) spectrum [5, 6]. According to the latest data report of China Antimicrobial Surveillance Network (CHINET, http://www.chinets.com/) in 2022, *A. baumannii* accounted for 7.5% of clinical isolates in China, ranking fifth among them [7]. The management of infected patients is difficult because of its complex drug-resistance mechanisms, the most worrying of which is carbapenem-resistant *A. baumannii* (CRAB). It is worth noting that the drug resistance rate of *A. baumannii* to antibiotics commonly used is basically increasing in China year by year. For example, the carbapenem-resistance rate of *A. baumannii* isolates such as imipenem and meropenem increased from 32.9% and 41.3% in 2005 to 77.7% and 79% in 2019, respectively. After 2019, the carbapenem-resistance rate decreased slightly, but remained above 70% [7].

According to the 2021 report from China Antimicrobial Resistance Surveillance System (CARSS, http://www.carss.cn/Report), he resistance rate of *A. baumannii* to carbapenem (imipenem or meropenem) was 54.3% on average, which was 0.6% higher than that in 2020 [8]. There are some differences in drug resistance rates among different regions. The average carbapenem-resistance rate of *A. baumannii* in Gansu province is 48.9% ranking in the middle level in China, which has also attracted our attention.

With the wide application and development of sequencing technology, many studies have focused on the genome-wide characteristics of *A. baumannii* isolates, including not only drug resistance and drug resistance genes, but also sequence types, plasmids, phylogenetic relationships and so on [9–12]. However, there are different characteristics in *A. baumannii* isolates from different hospitals.

Our study first analyzed the distribution and drug resistance characteristics, then clarified genomic characteristics of *A. baumannii* isolates from a hospital in Gansu province through whole-genome sequencing (WGS) technology, which could help guide infection control measures, prevention, and targeted antimicrobial therapy.

## Method

### Bacterial classification and antimicrobial susceptibility testing

Sixty-two non-repeated isolates were collected from Qingyang people's hospital in Gansu province, China, from August 2015 to November 2021. Bacterial isolates were initially subjected to identification using the VITEK-2 Compact system. Subsequently, the phenotypic identification was corroborated by whole-genome sequencing (WGS), where the genomic data obtained were compared against reference databases to validate and refine the taxonomic classification at the species or subspecies level. Antimicrobial susceptibility test to common clinical antibiotics was also detected by VITEK-2 compact system. Except the minimum inhibitory concentration (MIC) value of polymyxin was determined by the broth microdilution method. *Escherichia coli* ATCC25922 was used as a quality control strain. Seventeen common antibiotics include ticarcillin/clavulanic acid, piperacillin/tazobactam, ceftazidime, cefoperazone/sulbactam, cefepime, aztreonam, imipenem, meropenem, amikacin, tobramycin, ciprofloxacin, levofloxacin, doxycycline, minocycline, tigecycline, polymyxin, compound sulfamethoxazole.

The antimicrobial susceptibility test results were interpreted according to the Clinical and Laboratory Standards Institute (CLSI, 2021 version, https://clsi.org) unified protocol. Defining MDR, XDR and Pandrug resistant (PDR) criteria according to an international expert proposal for interim standard definitions for acquired resistance [13].

### Whole-genome sequencing

Genomic DNA was extracted from bacterial cultures using the QIAamp DNA Mini Kit (Qiagen, Hilden, Germany) according to the manufacturer’s instructions. DNA concentration and purity were measured using Qubit 4.0 (Thermo Fisher Scientific) and Nanodrop One (Thermo Fisher Scientific) at the same time. Sequencing libraries were generated using NEB Next Ultra DNA Library Prep Kit for Illumina (New England Biolabs) following the manufacturer's recommendations. The library quality was assessed on the Qubit 4.0 Fluorometer (Life Technologies) and Qsep400 High-Throughput Nucleic Acid Protein Analysis System (Houze Biological Technology Co.). DNA libraries were constructed with 150-bp paired-end fragments and sequenced on the HiSeq 2500 sequencer at Novogene Company (Beijing, China).

### Bioinformatics analysis

Sequencing reads were quality filtered using the FastQC v0.11.8 software [14], adapters and low-quality reads were removed and filtered out using Trimmomatic with default parameters [15]. The *A. baumannii* strain XH731 (NZ_CP021321.1) was selected as the best-matching chromosomal reference by the genomic distance estimation tool Mash v2.3 [16]. Assembly of sequencing reads was carried out with Unicycler v0.4.8 in normal mode [17]. Then the quality of the acquired genome was assessed by QUAST v5.2.0 [18]. Genome assemblies was annotated by Prokka v1.12 [19]. Then a core-genome alignment was derived by Panaroo v1.2.10 [20]. GTR + I + G4 was selected as the best evolutionary model by using Modeltest-ng v0.1.7 [21]. The maximum likelihood phylogenetic tree was constructed by IQ-TREE v2.2.2 with 1000 bootstrap replicates [22]. The tree was midpoint-rooted and visualized by Figtree v1.4.4 (http://tree.bio.ed.ac.uk/software/figtree/).

Sequence types (STs) and unknown STs were verified by MLST v2.23.0 [23] and MLST server v2.0 via the online service of the Center for Genomic Epidemiology of the Danish University of Technology (CGE) [24] respectively. The MLST analysis of these two software was based on the Institute Pasteur MLST schemes. The capsular polysaccharides loci (KL) and lipooligosaccharides outer core loci (OCL) were detected using the *A. baumannii* KL and OCL reference sequence databases by Kaptive v2.0.6 [25]. Resistance genes was identified with Abricate v0.8 (https://github.com/tseemann/abricate) using resfinder database [26]. The genotypic structure surrounding the special resistance gene was annotated by RAST (http://rast.nmpdr.org/) [27] and Easyfig v2.2.5 [28] was used to visualize the multiple sequence alignment. As a set of modular tools, the MOB-suite was used to reconstruct and type plasmids [29].

## Results

### General features of the patients and *A. baumannii* isolates

From August 2015 to November 2021, *A. baumannii* isolates was obtained from 62 samples including catheter (1/62), cerebrospinal fluid (1/62), urine (1/62), skin (1/62), wound secretion (3/62), sputum (54/62) and pleural effusion (1/62). Isolates were obtained from 47 (75.8%) men between 36 and 81 years old and 15 (24.2%) women between 12 days and 70 years old. The wards mainly include the intensive care unit (ICU), infection ward, orthopedic ward, respiratory ward, surgery ward, neonatal ward and traditional Chinese medicine ward. The detailed clinical information from patients with *A. baumannii* isolates was shown in Supplementary Table S1.

### Antimicrobial susceptibility testing

As shown in Table 1, 62 *A. baumannii* isolates were resistant to aztreonam (100%). Most isolates (56/62) were resistant to multiple drugs. Most isolates were resistant to piperacillin/tazobactam (88.71%), ceftazidime (88.71%), imipenem (85.48%), meropenem (85.48%), ciprofloxacin (82.26%), ticarcillin/clavulanic acid (80.65%), cefepime (79.03%), doxycycline (77.42%) and tobramycin (74.19%), but most isolates were sensitive to tigecycline (96.87%), polymyxin (95.16%) and minocycline (79.03%). Among 62 *A. baumannii* isolates, 55 MDR and 1 XDR *A. baumannii* isolates were identified, respectively. The MIC values of the 62 isolates were provided in Supplementary Table S2.

**Table 1:** Antimicrobial susceptibility of *Acinetobacter baumannii* Isolates

Antimicrobials	Resistant n (%)	Intermediate n (%)	Susceptible n (%)
Amikacin	24 (38.70)	2 (3.23)	36 (58.06)
Tobramycin	46 (74.19)	2 (4.83)	14 (22.58)
Aztreonam	62 (100.00)	0 (0.00)	0 (0.00)
Polymyxin	3 (4.84)	0 (0.00)	59 (95.16)
Compound Sulfamethoxazole	31 (50.00)	0 (0.00)	31 (50.00)
Ciprofloxacin	51 (82.26)	3 (4.84)	8 (12.90)
Meropenem	53 (85.48)	0 (0.00)	9 (14.52)
Minocycline	4 (6.45)	9 (14.52)	49 (79.03)
Doxycycline	48 (77.42)	4 (6.45)	10 (16.13)
Piperacillin/tazobactam	55 (88.71)	0 (0.00)	7 (11.29)
Tigecycline	1 (1.61)	1(1.61)	60 (96.78)
Ticarcillin/clavulanic acid	50 (80.65)	5 (8.06)	7 (11.29)
Cefepime	49 (79.03)	7 (11.29)	6 (9.68)
Cefoperazone/sulbactam	23 (37.10)	26 (41.94)	13 (20.96)
Ceftazidime	55 (88.71)	0 (0.00)	7 (11.29)
Imipenem	53(85.48)	0(0.00)	9(14.52)
Levofloxacin	17(27.41)	38(61.3)	7(11.29)

### Genomic characterization of *A. baumannii*

The genome sequences of 62 *A. baumannii* were obtained by whole-genome sequencing (Supplementary Table S3). Reads mapping to *A. baumannii* reference genome XH731 showed an average of 97.8% coverage among all 62 genomes. Seven sequence types were identified (Fig. 2 and Supplementary Table S1). Most of these *A. baumannii* isolates belonged to ST2 (52/62), which mainly isolated from ICU (27/52) and neurosurgery ward (12/52). ST2 *A. baumannii* isolates isolated in 2015, 2016 and 2021, with the largest number in 2016 (29/52). Others belonged to ST34 (1/62), ST40 (3/62), ST104 (1/62), ST132 (2/62) ST1108 (1/62), and ST1376 (2/62).

Thirteen distinct KL (KL3, KL161, KL230, KL47, KL61, KL72, KL106, KL160, KL22, KL33, KL34, KL45, KL9) configurations and five OCL (OCL1, OCL6, OCL21, OCL15, OCL18) configurations were detected among the isolates (Fig. 2 and Supplementary Table S1). The most common KL loci and OCL loci were KL3 (38/62) and OCL1(54/62), respectively.

### Phylogenetic analysis

**Fig. 1:**
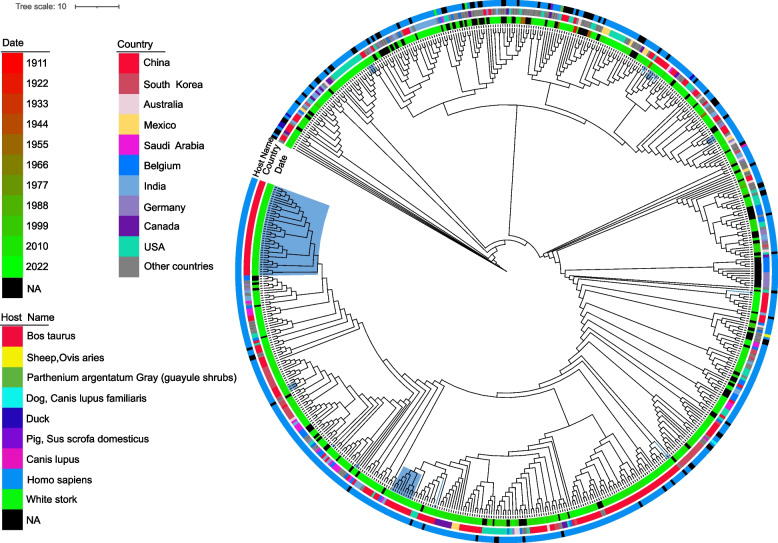
Phylogenetic analysis map of 629 *A. baumannii* isolates. The phylogenetic tree contains 567 *A. baumannii* isolates (ab_number) from BV-BRC and 62 *A. baumannii* isolates in this study (gs_number), annotating the isolation time, country, and host origin of the *A. baumannii* isolates from the inner ring to the outer ring. The black block means NA (Not available). The blue blocks on the tree branches signifies 62 *A. baumannii* isolates in this study

In order to explore the relationship between the *A. baumannii* isolates collected in this study and those previously studied, genome sequences of 567 *A. baumannii* isolates (Supplementary Table S4) were downloaded from the Bacterial and Viral Bioinformatics Resource Center (BV-BRC, https://www.bv-brc.org/), which were combined with 62 isolates in this study for the phylogenetic analysis. Figure 1 showed that the 62 isolates in this study did not cluster completely by source, but were dispersed among multiple clades in the common genome. Among the 52 ST2 strains collected in this study, phylogenetic analysis revealed that despite being isolated from the same hospital, they did not cluster on the same branch. Instead, they formed two main branches, comprising 38 and 9 isolates respectively, with the remaining 5 isolates scattered across different branches.

### Antibiotic resistance gene profiles of *A. baumannii*

**Fig. 2:**
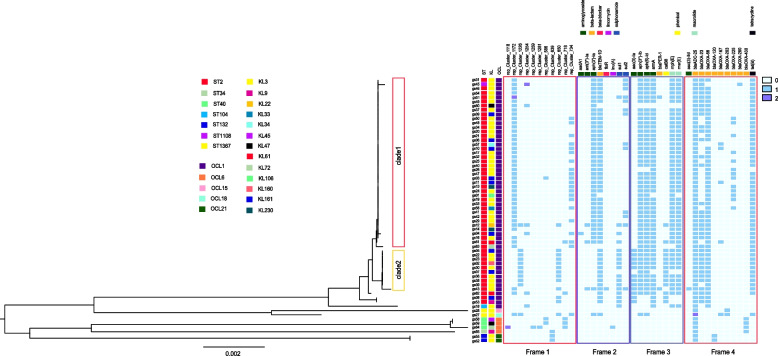
The phylogenetic tree and distribution of STs, KL types, OCL types, plasmids and ARGs among 62 *A. baumannii* isolates. On the left half, a phylogenetic tree was generated using IQ-TREE v2.2.2 software and midpoint-rooted for improved visualization using Figtree v1.4.4. Isolates are color-coded according to their STs, capsular polysaccharide (KL) types, and lipooligosaccharides outer core loci (OCL). Fifty-two ST2 and one ST1108 isolates were clustered into two monophyletic clades, referred to as Clade1 and Clade2. On the right, four frames provide information about plasmid carriage and distribution of Antibiotic Resistance Genes (ARGs). Frame1 indicates the distribution of plasmids; Frame 2 highlights ARGs found only on plasmid contigs; Frame 3 focuses on ARGs present on both plasmid and chromosome contigs; Frame 4 shows ARGs exclusive to chromosome contigs, the light blue squares represent the absence of plasmid or ARGs, dark blue and purple squares represent one and two plasmids or ARGs, respectively

The distribution of antibiotic resistance genes (ARGs) of different STs *A. baumannii* isolates was shown in Fig. 2. A total 27 types of ARGs were identified, conferring resistance to 7 classes of antibiotic including aminoglycosides, beta-lactams, lincomycin, sulphonamide, phenicols, macrolides, tetracycline. Among them, *bla*_TEM-1D_ (51/61) and *bla*_ADC-25_ (62/62) genes were the most prevalent on the putative plasmid and chromosomal contigs, respectively. The *bla*_ADC-25_ gene only on the chromosomal contigs was further explored carried by all strains.

**Fig. 3:**
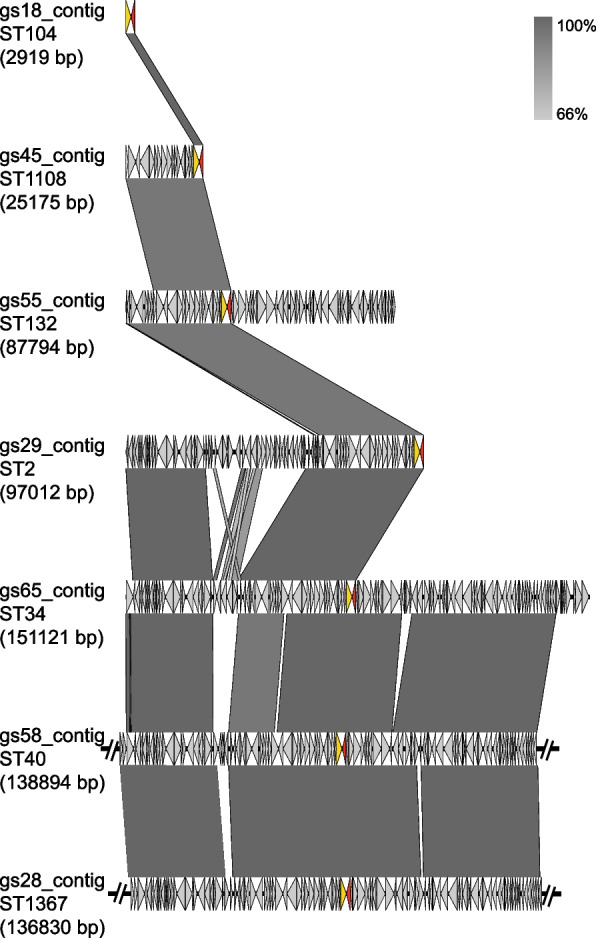
Analysis of *bla*_ADC-25_ surrounding environment. Each arrow represents an open reading frame (ORF). The red arrows represent *bla*_ADC-25_ gene, the orange arrows represent putative outer membrane protein and the gray arrows represent hypothetical proteins and others. The grey shaded areas represent highly homologous regions between contigs. The two overly long contigs of gs58 and gs28 have been truncated to improve the figure visualization

In order to understand the surrounding environment of *bla*_ADC-25_ gene, we randomly selected a representative isolate of each ST type. Then the *bla*_ADC-25_-containing contig sequences were extracted from the genome data, among which seven contigs from isolates gs18, 28, 29, 45, 55, 58 and 65. However, no mobile genetic element, such as a transposon or insertion sequence (IS), was found surrounding the *bla*_ADC-25_, as shown in Fig. 3.

### Plasmid carriage profile

Plasmids were detected in 61 of 62 *A. baumannii* isolates (98.4%), totaling 110 putative plasmids (median: 2 per isolate; range: 1–5). The putative plasmids were grouped into 11 clusters by mob suite, including rep_cluster_586, rep_cluster_639, rep_cluster_650, rep_cluster_734, rep_cluster_718, rep_cluster_1118, rep_cluster_1172, rep_cluster_1226, rep_cluster_1254, rep_cluster_1259, rep_cluster_1281. *A. baumannii* ST2 isolates mainly carried rep_cluster_1172 (40/52) and rep_cluster_734 (20/52). In addition, no virulence genes were found in the 110 putative plasmid contigs.

## Discussion

Phylogenetic analyses combined with BV-BRC and the data in this study showed that the 629 isolates were derived from 34 countries, with most of them being from Asia. A review summarizes *Acinetobacter* accounts for approximately 2% of nosocomial infections in the USA but these rates are doubled in Asia and the Middle East [30]. *A. baumannii* infection in Asia is a considerable challenge to healthcare professionals in hospitals.

According to the source distribution of 62 *A. baumannii* isolates in this study, the main specimen type was sputum, which is consistent with the results of previous studies [31, 32]. The wards distribution showed that the isolates mainly from ICU and neurosurgery ward. Especially for patients in the ICU, the establishment of artificial airway operations such as ventilator invasion may form biofilms on the surfaces, thus increasing the risk of infection and antibiotic resistance of *A. baumannii* [2, 33–35]. Therefore, patients in the ICU and neurosurgery ward are more infected with *A. baumannii* and medical staff should pay attention to the standardization of invasive operations.

Although previous studies have shown that *A. baumannii* can produce wide-spectrum beta-lactamases, which affected the resistance of *A. baumannii* to β-lactam antibiotics by hydrolysis of beta-lactams [36, 37]. However, as wide-spectrum antibiotics, carbapenem antibiotics are widely used in clinic treatment, causing high drug resistance. In this study, *A. baumannii* isolates showed high-level resistance to carbapenem antibiotics and high-level sensitivity to tigecycline, polymyxin and minocycline, which was similar to the results from Italy [38] and China [39]. Therefore, carbapenem antibiotics in combination with polymyxin or tigecycline should be considered to in clinical treatment of MDR *A. baumannii* infection [40]. If there are co-infections, polymyxin will be used to treat patients [41, 42].

Seven STs in the study belonged to known *A. baumannii* international clones (ICs). The analysis of all publicly available genome sequences in 2019 indicated that ST2, ST1, ST79 and ST25 account for more than 71% of all sequenced genomes, with ST2 by far the most dominant type [43]. The results of MLST typing of 62 *A. baumannii* isolates showed that ST2 accounted for 83.9% (52/62), which was the dominant ST type in this study. The results are consistent with other related researches in China [44, 45]. The reason why ST2 isolates have become the dominant ST type may be due to its strong biofilm formation ability, high serum resistance, and high pathogenicity [45].

Several studies have proved that the prevalence of *A. baumannii* is associated with *bla*_ADC-25_ [12, 44], *bla*_OXA-23_ and *bla*_OXA-66_ genes [46]. This has also been proved in our study, which exhibited all *A. baumannii* isolates have *bla*_ADC-25_ gene, which are dominant in cephalosporins and carbapenems resistance in *A. baumannii* ST2 isolates. Insertion sequences (ISs) was responsible for the overexpression of the chromosomally encoded *bla*_ADC_ gene [47]. There are no ISs in the *bla*_ADC-25_ surrounding environment in the selecting representative isolates per ST, suggesting that the gene is an inherent drug resistance gene located on chromosomes.

The MOB-suite identified contigs of plasmid origin showed both high sensitivity and specificity (95 and 88%, respectively) [27]. Therefore, we used the software for clustering, reconstruction and typing of plasmids from assemblies. Unfortunately, these rep_clusters are not identified to specific known replicators. But our focus is mainly to understand the plasmids sequences for ARGs-carrying contigs.

The phylogenetic analysis of the 62 strains revealed that among the 52 ST2 strains, two distinct monophyletic clades were formed on the phylogenetic tree. Clade1 predominantly harbored the rep_cluster_1172 plasmid along with the *aph* (3')-Ia resistance gene, whereas clade2 was characterized by the presence of the rep_cluster_1226 plasmid and lacked the *aph* (3')-Ia resistance gene. This finding illuminated the underlying relationship between different genetic lineages and their associations with specific plasmids as well as antibiotic resistance profiles.

There are some limitations in this study. The number of other types of specimens apart from sputum was very small. The number of specimens such as pleural effusion and urine can be increased in follow-up study. Although we used the cluster codes provided by MOB-cluster for description of plasmids that share significant sequences without the need for defined biomarkers. Only rep_clusters were obtained which cannot be identified to specific known replication factors. We mainly focused on studying resistance gene carrier, and virulence and detailed transmission routes of *A. baumannii* isolates need to be understand in the future.

To sum up, *A. baumannii* isolates in this study are resistant to most antibiotics and carries a variety of drug resistance genes, indicating the severe situation of drug resistance. The surveillance of drug resistance and active screening of *A. baumannii* should be strengthened for curbing the spread of *A. baumannii* in hospitals. There are few previous studies about phenotypic and molecular characteristics of *A. baumannii* in Gansu province. Our study provided implications to further explore the main genotypic and phenotypic characteristics of *A. baumannii* in Gansu province.

## Conclusions

Our study reveals genotypic and phenotypic characteristics of *A. baumannii* isolates from a hospital in Gansu Province, China, offering reference into further exploring the main characteristics of *A. baumannii*.

### Supplementary Information

Supplementary Material 1. Supplementary Table S1. Detailed clinical information from patients with A. baumannii isolates. (1) Only the gs65 patient is 12 days old; (2) ICU: intensive care unit. Supplementary Table S2. Antimicrobial susceptibilities of sixty-two Acinetobacter baumannii isolates. R: Resistant; I: Intermediate; S: Susceptible. Supplementary Table S3. Quality assessment information for genome assemblies. Supplementary Table S4. Basic information of 567 Acinetobacter baumannii isolates from BV-BRC. NA means not available.

## Data Availability

Data is deposited in National Microbiology Data Center (NMDC) with accession numbers NMDC10018764 (https://nmdc.cn/resource/genomics/project/detail/NMDC10018764).

## Abbreviations

*A. baumannii*: *Acinetobacter baumannii*
MLST: Multilocus sequence type
WHO: World Health Organization
MDR: Multidrug resistance
XDR: Extensive drug resistance
CHINET: China Antimicrobial Surveillance Network
CRAB: Carbapenem-resistant *A. baumannii*
CARSS: China Antimicrobial Resistance Surveillance System
WGS: Whole-genome sequencing
MIC: Minimum inhibitory concentration
CLSI: Clinical and Laboratory Standards Institute
PDR: Pandrug resistant
STs: Sequence types
CGE: Genomic Epidemiology of the Danish University of Technology
KL: Capsular polysaccharides loci
OCL: Lipooligosaccharides outer core loci
BV-BRC: Bacterial and Viral Bioinformatics Resource Center
ARGs: Antibiotic resistance genes
ICs: International clones
